# Myasthenia gravis with achalasia secondary to thymoma: a case report and literature review

**DOI:** 10.1186/s41983-023-00636-4

**Published:** 2023-03-15

**Authors:** Nourelhoda A. Haridy, Eman M. Khedr, Asmaa M. Hasan, Ahmed A. Maghraby, Essam Abdelmohsen, AbdelHamid M. Aly

**Affiliations:** 1 Department of Neurology and Psychiatry, Faculty of Medicine, Assiut University, Assiut, Egypt; 2 Department of Internal Medicine, Gastroenterology Unit, Faculty of Medicine, Assiut University, Assiut, Egypt

**Keywords:** Myasthenia gravis, Thymoma, Dysphagia, Achalasia, Case report, High-resolution esophageal manometry

## Abstract

**Background:**

Myasthenia gravis is an autoimmune neuromuscular junction disorder characterized by fatigable muscle weakness and autoantibodies. Frequent associations exist between myasthenia gravis and thymic abnormalities, including hyperplasia and thymoma. Several autoimmune illnesses have been identified to be associated with thymoma; however, a few case reports have linked thymoma and achalasia, and the underlying mechanism is unknown.

**Case report:**

A 43-year-old man with thymoma-associated myasthenia gravis presented with dysphagia that was refractory to conventional treatment of myasthenia gravis. This dysphagia was challenging to diagnose even after multiple gastroenterology consults and upper endoscopy. The diagnosis of achalasia type II was established after a comprehensive evaluation, including upper endoscopy, barium swallow, and high-resolution esophageal manometry. The patient underwent elective pneumatic balloon dilatation, which successfully alleviated his dysphagia.

**Conclusion:**

This case confirmed the association between myasthenia gravis secondary to thymoma and achalasia and showed how the diagnosis of achalasia was challenging. Awareness of this association is crucial for early diagnosis and treatment, improving affected patients’ quality of life.

## Background

Myasthenia gravis (MG) is an autoimmune neuromuscular junction disorder characterized by fatigable muscle weakness and the presence of autoantibodies, with acetylcholine receptor (AChR) antibodies presented in up to 80% of individuals with generalized MG [1]. MG commonly includes the extraocular muscles, causing diplopia and/or ptosis. Generalized MG affects the face, axial, limb, bulbar, and respiratory muscles. MG is often linked with thymic abnormalities, including hyperplasia and thymoma; thymus imaging is suggested at presentation [2].

Thymomas, the most prevalent primary anterior mediastinal mass, are rare primary mediastinal tumors arising from the thymic epithelium [3]. Approximately one-third of thymoma cases are identified during a radiographic assessment for an autoimmune disease, most commonly MG [4]. Other autoimmune diseases linked to thymoma and discovered during or after thymectomy included systemic lupus erythematosus, inflammatory myopathies, thyroid abnormalities, and others [5, 6]. Achalasia was detected among these autoimmune conditions [7]. Achalasia is a rare esophageal motility condition characterized by abnormalities in the esophageal body’s motility and the relaxation of the lower esophageal sphincter (LES) [8, 9]. Achalasia, which can cause severe dysphagia, substantially impacts patients’ quality of life (QoL) and can be challenging to detect and treat [10].

In humans, the coexistence of MG with achalasia or megaesophagus has been established infrequently [11]; nevertheless, this coexistence is common in animals such as dogs, cats, and rodents [11, 12]. A few case reports described the association between MG and achalasia [13–15] and the association of thymoma with paraneoplastic MG and achalasia [16, 17]. Herein, we describe the first Egyptian case report that suffered from MG secondary to thymoma and associated with dysphagia with food regurgitation due to achalasia.

## Case presentation

A 43-year-old male patient, a cigarette smoker who works as an office employee, was diagnosed with MG in February 2019. The condition started on December 2018 with weakness in the left side of his face associated with dysarthria that was misdiagnosed as left Bell’s palsy, for which the patient received steroid therapy and physiotherapy with no response to the medication. A few months later, the condition progressed to be associated with diplopia with a blurring of vision, followed by bulbar manifestations in the form of dysphagia with nasal regurgitation to fluid and frequent choking. Within a few days, the patient developed neck muscle weakness, especially flexion, with proximal weakness of the upper and lower limbs. These manifestations had diurnal variation, with increased symptoms at the end of the day and increased with exertion. Till this time, the patient received non-specific treatment.

The patient sought medical advice, and repetitive nerve stimulation in the frontalis and deltoid muscles was done in February 2019, revealing a positive decrement test for myasthenia. The serum level of the acetylcholine antibody was positive, and the titer was 7.9 nmol/l (negative if < 0.4 nmol/l). The patient was hospitalized and received six sessions of plasmapheresis with complete improvement. During this time, no chest imaging was done to assess the thymus gland status and the patient was managed conservatively and was prescribed acetylcholine esterase inhibitors (pyridostigmine 60 mg) twice daily with azathioprine twice daily and steroids that were gradually tapered, but the patient was incompliant with the treatment and developed recurrent attacks of exacerbations.

In September 2021, the patient complained of epigastric pain, dysphagia, and repeated food regurgitation and sought medical advice several times. Upper endoscopy revealed only hyperemic edematous mucosa with multiple fundal and pre-pyloric gastric ulcers. The patient was prescribed medical treatment with proton pump inhibitors with no improvement in his gastrointestinal tract (GIT) symptoms (vomiting and dysphagia, described as a sensation of food sticking behind his chest).

**Fig. 1:**
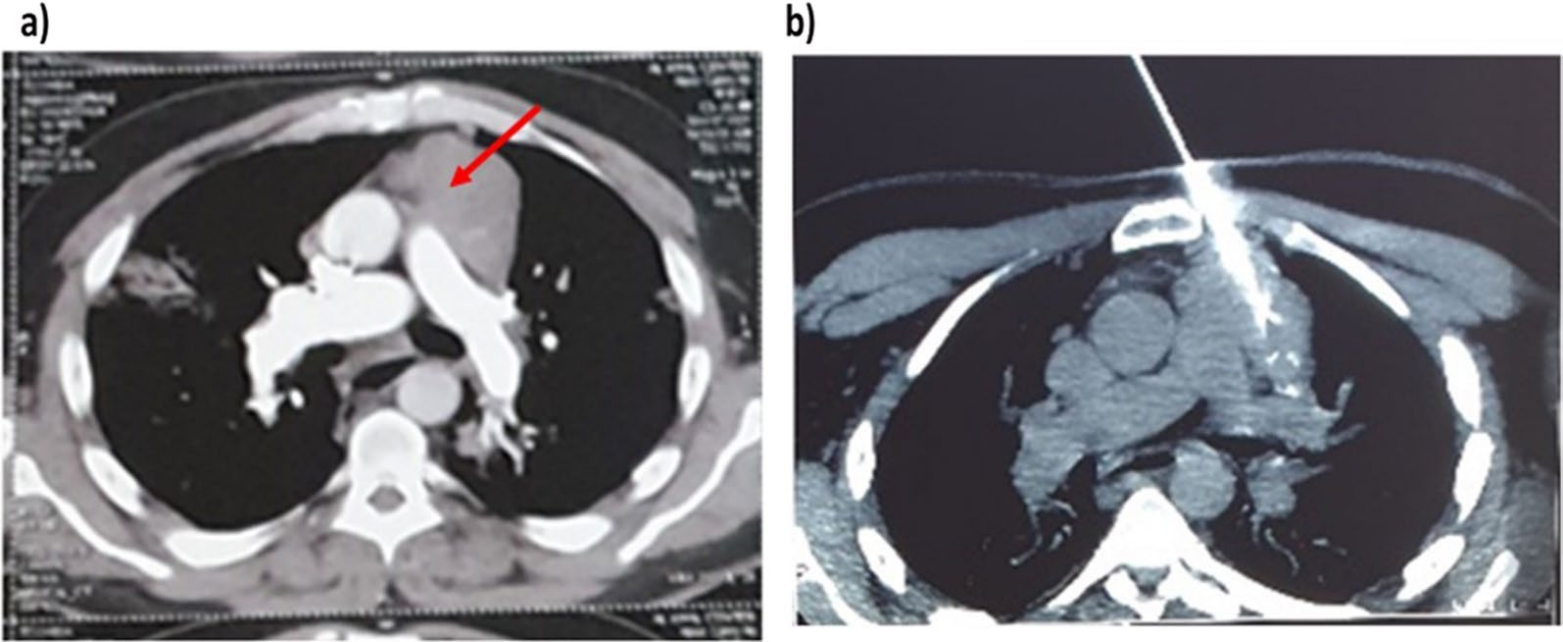
**a** Red arrow in the axial view of the chest’s computed tomography showing thymoma. **b** True cut needle biopsy

In November 2021, the patient developed worsening of his MG symptoms with a chest infection suggestive of pneumonia and was admitted to the hospital’s intermediate care unit. The patient was investigated for Corona Virus Disease 2019 (COVID-19) infection with a complete metabolic profile, and multislice computed tomography (MSCT) chest were done. The complete metabolic profile was normal, apart from absolute lymphopenia. The MSCT chest with and without contrast showed a ground glass appearance in both lungs suggestive of COVID-19 (CORAD III). Other findings in the MSCT chest included: iso-dense left anterior mediastinal soft tissue mass measuring about 6.7 × 6.7 × 5 cm, with minute foci of calcification. The mass showed homogenous enhancement in the post-contrast study, partially encasing the left innominate vein with no definite infiltration (Fig. 1a). Also, the distal part of the esophagus was minimally dilated and filled with fluid with no definite mass lesion. At first, the patient was managed for his COVID-19 infection. In January 2022, after controlling the infection, CT guided true cut needle biopsy (Fig. 1b) was done for his left anterior mediastinal mass, which revealed undifferentiated neoplasm for immunohistochemistry.

A few days later, the patient was transferred to neurology due to his myasthenic crisis. His neurological examination showed bilateral ptosis, diplopia, weakness of muscle of facial expression, neck muscle weakness more in neck flexors, and proximal weakness of both upper and lower limbs (grade 4a according to the medical research council muscle grading system), with unremarkable rest of neurological examination. The patient received five sessions of plasmapheresis with complete improvement apart from unexplained persistent dysphagia and vomiting. The patient was assessed by a cardiothoracic consultant and prepared for elective thymectomy. In March 2022, transsternal extending thymectomy of left thymus mass with partial resection of innominate vein via median sternotomy. The histopathological examination revealed a picture of thymoma type B2, p T1 (cortical thymoma and polygonal cell thymoma). After surgery, the patient was stable because he did not need postoperative mechanical ventilation and did not develop a postoperative myasthenic crisis. The patient was discharged home and received the postoperative treatment (levofloxacin, bronchodilators and anti-inflammatory) and continued MG medication (pyridostigmine 60 mg four times daily).

**Fig. 2:**
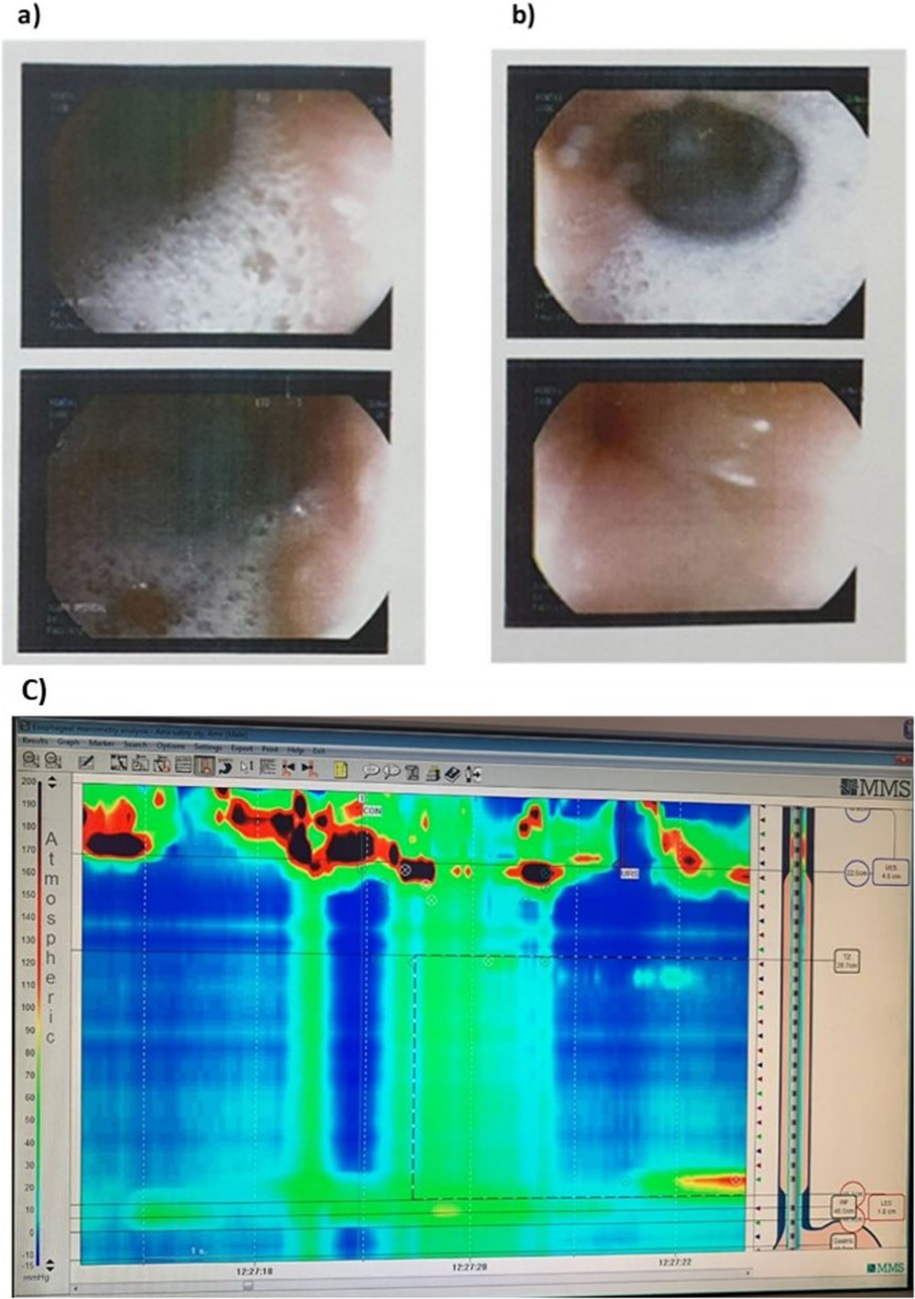
**a** Upper gastrointestinal endoscopy showing dilated esophagus and retained fluids. **b** Tight lower esophageal sphincter. **c** HRM of achalasia type II: showed the failure of LES relaxation, high resting LES pressure, and absent esophageal peristalsis (100%) with panesophageal pressurization to greater than 30 mmHg

**Fig. 3:**
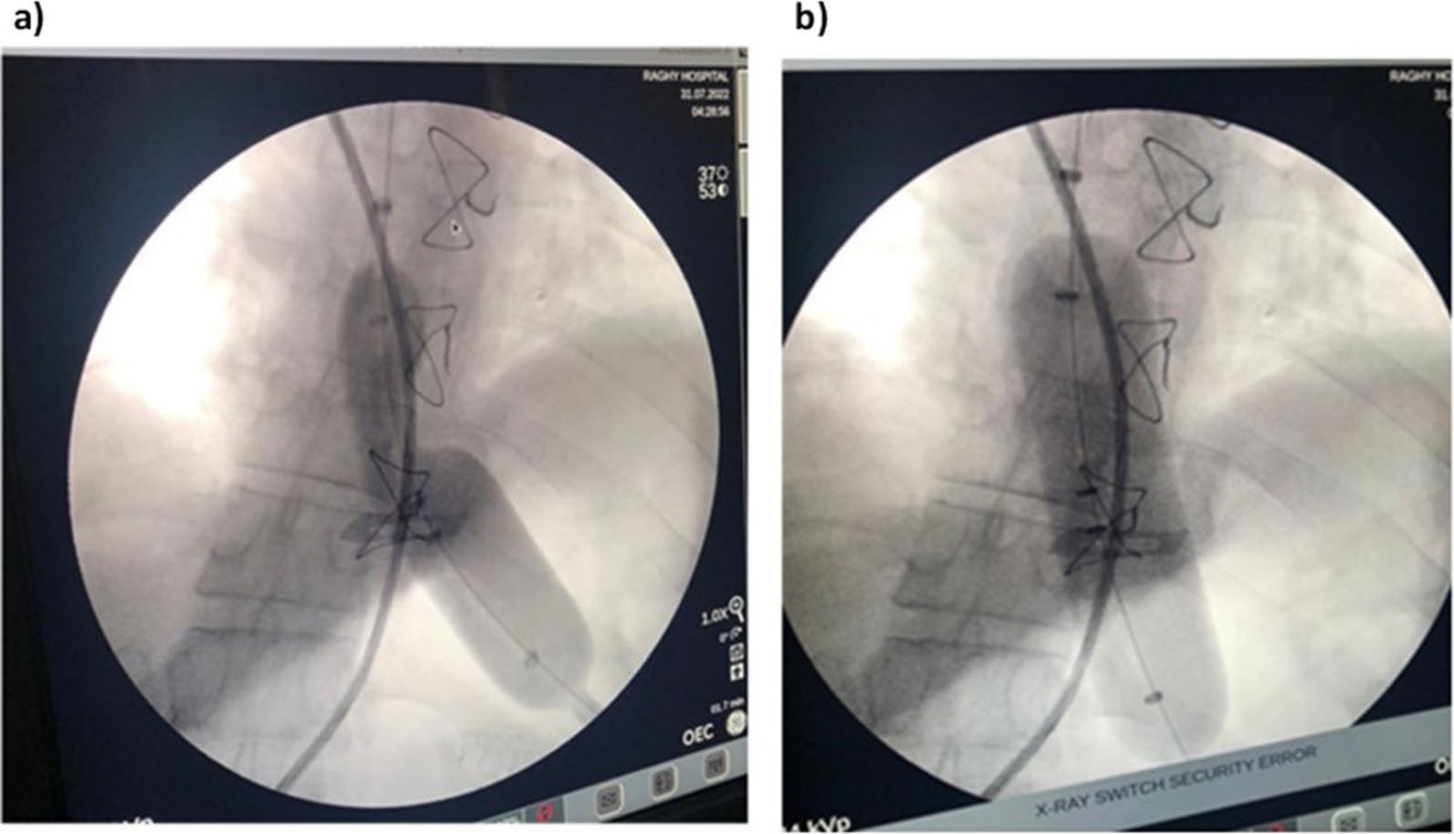
Fluoroscopic image of achalasia using a 3.5-cm pneumatic dilator showing: **a** pneumatic balloon dilation with subsequent dilation to obliterate the balloon waist; **b** after successful pneumatic balloon dilation with balloon inflation to the maximum pressure of 13 mmHg

In June 2022, another attack of MG exacerbation for which the patient was admitted and received five sessions of plasmapheresis with partial improvement of his symptoms except for persistent vomiting after meals and regurgitation of undigested food with dysphagia. The patient was discharged on medical treatment (pyridostigmine 60 mg three times daily, azathioprine 50 mg twice daily, and steroid with gradual tapering). After gastroenterology consultation for his GIT symptoms and investigations, including upper endoscopy, barium swallow, and high-resolution esophageal manometry (HRM), the diagnosis of achalasia was confirmed. Upper endoscopy showed dilated esophagus with retained fluid and spastic cardia (Fig. 2a, b). The barium swallow study showed dilated esophagus with a bird beak appearance, and HRM showed achalasia type II (Fig. 2c). In July 2022, elective pneumatic balloon dilatation (PBD) (Fig. 3a, b) was done with successful dilation and remarkable improvement of the patient’s GIT symptoms. The patient is now stable on his MG medical treatment for the sixth month’s follow-up.

## Conclusions

A 43-year-old male patient with MG and thymectomy complained of persistent vomiting and dysphagia after swallowing each meal that did not respond to standard MG therapy, including thymectomy and rapid immunomodulatory therapy (plasmapheresis). These GIT symptoms necessitated gastroenterology consultations several times that finally succeeded in adequately diagnosing the associated condition, achalasia type II, and prescribed the proper therapy, including pneumatic balloon dilatation with a complete improvement of the patient’s symptoms.

The current case report demonstrates the association of thymoma with MG and achalasia. This case replicates the previous finding reported by other studies [16, 17] that described two cases with a similar association of the three conditions with different ages of presentation. In other case reports, thymoma was associated with neuromuscular conditions, such as megaesophagus, without any neurological evidence of MG [7]. Moreover, MG was reported to be associated with megaesophagus without thymoma [11] or MG with achalasia without thymoma [13–15]. Furthermore, achalasia could be a complication of radiotherapy for thymoma that cause MG [18]. The cases of MG with achalasia with and without thymoma [11, 13–17] are summarized in Table 1.

**Table 1:** A literature review of case reports with MG and achalasia with and without thymoma

	Kaminski (1999) [16]	Kornizky et al. (2000) [13]	Rison (2009) [14]	Desuter et al. (2015) [11]	Seabi and Mokgoko (2020) [17]	Vinod (2020) [15]	The current study
Gender and age	F/ 75	F/ 69	M/ 83	M/ 79	M/ 35	M/ 11	M/ 43
Ethnicity	Unknown	Unknown	USA	Unknown	Unknown	Indian	Egyptian
GIT symptoms	Food sticking in the upper chest and regurgitation of undigested food	Dysphagia and occasional non-specific retrosternal pain	Painless secretions in the back of his throat inhibit swallowing	Aphagia, followed by nasogastric feeding, then percutaneous endoscopic gastrostomy	Dysphagia	Persistent vomiting, difficulty swallowing, and regurgitation	Dysphagia (food sticking in the upper chest and regurgitation of undigested food)
Other associated conditions	No	Polymyositis	DM2, arthritis, cervical spinal stenosis. Past peptic ulcers with a previous gastrointestinal bleed secondary to past steroid use. Left nephrectomy secondary to prior staghorn calculi, previous lumbar spine surgery, previous cholecystectomy, and previous bariatric surgery	No	No	No	No
Acetylcholine antibody titer	4.4 nmol/ L (negative if < 0.4 nmol/l)	Positive	7.94 nmol/ L (negative if < 0.4 nmol/l)	N/A	N/A	0.69 nmol/l (serum reference value < 0.25 nmol/l)	7.9 nmol/l (negative if < 0.4 nmol/l)
CT chest	Unremarkable	Unremarkable	Unremarkable	Unremarkable	N/A	Dilated esophagus	Anterior mediastinal soft tissue mass iso-dense in the non-contrast study with minute foci of calcification with homogenous enhancement in the post-contrast study
Treatment of MG	Anticholinesterase medication Six session PE	Pyridostigmine	Pyridostigmine (60 mg PO q 4 h) and azathioprine (50 mg PO bid, for the last 8–9 years). PE in case of MG crisis	Anticholinesterase medication	Anticholinesterase inhibitor and immunosuppression with prednisone and azathioprine	Steroids, Acetylcholinesterase inhibitor (pyridostigmine), and nifedipine	Acetylcholinesterase inhibitor (pyridostigmine). Immunosuppression with prednisolone and azathioprine. PE in case of MG crisis
Thymoma/type	Yes/ resection of mixed epithelial and lymphocytic thymoma one year before the symptoms Recurrent thymoma and carcinoid tumor in the lung	No thymoma	No thymoma	No thymoma	Yes/ resection of thymoma in 2017	No thymoma	Yes/ resection of thymoma in 2022. Thymoma type B2, p T1
Esophageal motility study	Absence of lower esophageal sphincter relaxation with multiple peristalses and simultaneous contraction of the body	Absent peristaltic waves in the esophageal body, with a normal pressure in the LES (17 mmHg, normal 15–30)	Not done	Not done	Not done	Failure of the lower esophageal sphincter to relax, elevated basal pressure, and peristalsis of the esophageal body suggestive of achalasia cardia	HRM showed achalasia type II. Failure of LES relaxation, high resting LES pressure, and absent esophageal peristalsis (100%) with panesophageal pressurization to greater than 30 mmHg
Barium swallow/esophageal transit study	Moderate esophageal dilatation	Marked dilated esophageal body with a beak-like narrowing of the terminal portion	Prominent cricopharyngeal sphincter with incomplete relaxation and no evidence of any cricopharyngeal bar	N/A	N/A	Dilated esophagus	Dilated esophagus with bird beak appearance
Endoscopy findings	Not done	Not done	Not done	Primary aspiration did not reveal regurgitation or UES blockage, but it did identify a significant lack of hypopharyngeal contraction	N/A	Dilated esophagus	Dilated esophagus with retained fluid and spastic cardia
Pathology/ histopathology	Loss of myenteric ganglion cells was confirmed/ no antibody binds to the Auerbach plexus	Not done	Not done	Not done	Not done		Not done
Management of dysphagia/ response to the treatment	Botulinum toxin injection Laparoscopic Heller myotomy. Improvement in swallowing and weight loss after the procedure	Prednisone and methotrexate. Some improvement in the dysphagia	Direct laryngoscopy followed by esophagoscopy with mechanical dilation of the cricopharyngeal sphincter. The patient’s swallowing markedly improved	Medical treatment of MG (pyridostigmine). The 4-year follow-up showed no deterioration in his oral feeding ability	N/A	Medical treatment for MG improves the dysphagia	Elective pneumatic balloon dilatation was successful and remarkably improved the patient’s symptoms

*CT* computed tomography, *DM2* diabetes mellitus type 2, *F* female, *GIT* gastrointestinal, *HRM* high-resolution esophageal manometry, *LES* lower esophageal sphincter, *MG* myasthenia gravis, *M* male, *N/A* not available, *PO* per oral, *PE* plasma exchange, *UES* upper esophageal sphincter

The current case had two important practical issues that required explanation; the first was the exacerbation of myasthenic symptoms 2.5 months after thymectomy despite the initial improvement, and the second was the delay in detecting the achalasia associated with MG related to thymoma.

Regarding the first issue, the response to thymectomy is variable, with some patients experiencing worsening and recurrence of MG symptoms or myasthenic crisis (MC) following thymectomy. Up to 50% of MG patients undergoing thymectomy, with an average follow-up duration of 7.0–8.9 years, have a relapse after achieving initial clinical responses [19]. This relapse of MG was identified to be related to several risk factors reported in earlier research [19–22]. Patients with thymoma were more likely to have a recurrence of their myasthenic symptoms after they reached complete stable remission and within a shorter period than in patients without thymoma [20], and this is more seen with type B2–B3 thymomas than those with A-B1 types according to the WHO histologic classification [21]. This could be explained by the fact that thymoma extrusion is inevitable during thymectomy, and auto-reactive T cells may be released from the thymoma into the peripheral circulation, resulting in autoimmunity and deterioration of MG status [23]. Other risk factors, including bulbar symptoms, generalized MG, and a history of previous MC, were identified as independent predictors of MG relapse after thymectomy [23]. The use of medications that are contraindicated in patients with MG, such as fluoroquinolones, can also exacerbate MG symptoms [24, 25].

All of these risk factors were present in the current patient, who had a history of thymoma type B2, GIT symptoms due to achalasia that could lead to infection, which is a trigger for developing relapse, a history of several exacerbations of his myasthenic symptoms, and postoperative treatment with levofloxacin (fluoroquinolone antibiotics).

Regarding the second issue, in the current case, the patient has complained of persistent dysphagia from September 2021 to June 2022, which means more than one and a half years to reach an accurate diagnosis. The patient was misdiagnosed as having pre-pyloric gastric ulcers as a cause for his vomiting. In this case, the delay in diagnosing achalasia could be explained by his MG, which is associated with dysphagia as bulbar muscle involvement, which could result in the regurgitation of food and fluid. Also, gastric ulcers could be related to the steroid therapy the patient received during his MG crisis. Accurate assessment of such cases is essential to avoid delay in diagnosis, which impacts the patient’s QoL and provide the appropriate intervention and treatment to improve the patient’s QoL.

Since achalasia still represents a challenging area for diagnosis and treatment in current practice [8], especially with non-professional gastroenterologists, accurate diagnosis requires a methodical diagnostic procedure. The diagnostic gold standard for achalasia is high-resolution esophageal manometry, which allows subtype definition and differentiation from other esophageal motor diseases [8, 26, 27]. The exact mechanism of achalasia is unknown [28, 29]; however, it was suggested that an autoimmune reaction targets esophageal myenteric neurons through cell-mediated and potential antibody-mediated processes in genetically predisposed patients is the potential mechanism of achalasia [9]. Moreover, it has been postulated that achalasia is not caused by a single mechanism but rather by a combination of infectious, autoimmune, and familial etiological factors. Recent evidence suggests that muscular eosinophilic esophagitis may have a role in the pathogenesis of achalasia in some patients [30].

The explanation of the association between thymoma and achalasia still needs to be determined. Some mechanisms have been proposed; Old report by Demos and colleagues proposed a hormonal relationship to be the cause of both illnesses, and the rationale for their assumption is the previous findings by Hasner and colleagues [31], who found that achalasia is more with benign thymoma, so achalasia in patients with thymoma is not limited to esophageal compression. Therefore, there may be more than a mechanical explanation for dysphagia in thymoma patients [7].

Another proposed mechanism is that the eosinophils may explain the connection between thymoma and achalasia caused by eosinophilic esophagitis. Eosinophils are innate immune system cells that primarily dwell within tissues and are most abundant in the GIT, excluding the healthy esophagus [32, 33]. Eosinophils only invade the esophagus during eosinophilic esophagitis, although their role is uncertain. In addition, eosinophils are also present in the human thymus, although their role in T cell development is unknown [32, 33]. T cell suppression is proposed as a potential function of eosinophils in eosinophilic esophagitis, and thymic eosinophils are demonstrated to be specialized cells capable of influencing thymocyte growth [33]. However, this mechanism needs confirmation by histopathological testing for the achalasia associated with thymoma. Depending on these assumptions, a common autoimmune mechanism, which still needs to be detected, could explain the association between the three conditions.

In conclusion, the current case confirmed the association between myasthenia gravis secondary to thymoma and achalasia. In addition, it has raised neurologists’ knowledge and awareness of achalasia in patients of MG complaining of recurrent vomiting and dysphagia resistant to standard therapy, which includes thymectomy and rapid immunomodulatory therapy. In addition, gastroenterologists, who may encounter a patient with MG who complains of dysphagia and is referred for an upper endoscopy, should consider this alternative diagnosis when examining and investigating the patient’s dysphagia.

## Data Availability

Not applicable.

## Abbreviations

AChR: Acetylcholine receptor
COVID-19: Corona Virus Disease 2019
GIT: Gastrointestinal tract
HRM: High-resolution esophageal manometry
MSCT: Multislice computed tomography
MG: Myasthenia gravis
MC: Myasthenic crisis
QoL: Quality of life
